# Abstract of Proceedings of the Buffalo Medical Association, Oct. 6th, 1874

**Published:** 1874-11

**Authors:** E. R. Barnes

**Affiliations:** Secretary


BUFFALO

### Medical and Surgical Journal.

VOL XIV. NOVEMBER, 1874. No. 4.

### Original Communications.

———:o:———

ART. I.—*Abstract of the Proceedings of the Buffalo Medical Association.* Buffalo, Oct. 6, 1874. Reported by E. R. BARNES, M. D., Secretary.

Members present, the Vice-President, Dr. Gould in the chair, and Drs. Miner, Johnson, Briggs, Hauenstein, Brecht, Harding, Brush, Boardman, Bartlett, Wyckoff, et. al.

The application for membership of Dr. Boysen was received, and he was invited to participate in the proceedings until his application could be acted upon.

After routine business Dr. MINER said that he had noticed, in the published transactions of a recent meeting of the Society, that there had been a discussion on a subject always interesting to a large number of the profession—that is, on venereal disease. All have opinions on this subject, some holding opinions peculiar to themselves, others according with the views of authors. While he would not renew an old discussion, he wished to refer to a case he had lately seen, which he thought remarkable. It was the communication by a man to his wife, of a gonorrhœa; the man himself not having the disease. The case did not admit of a suspicion of error. A gentleman of high respectability was led astray in a neighboring city, and had sexual connection. After the act he washed, and returning home the same day, he held intercourse with his wife. A few days after she had specific gonorrhœa. There is no doubt that the husband communicated the disease, yet he had himself not the slightest evidence of disease. The virus might have been conveyed on the outer folds of the foreskin. Such an occurrence is exceedingly rare, but it is possible, as we see the virus conveyed to the eye through the medium of a towel.

With reference to the specific virus of different venereal diseases, a question which was discussed at the meeting referred to, when the belief was expressed by some, that secondary symptoms might ensue after either a soft or a hard chancre, he wished to state his adherence to the theory of the duality of the syphilitic virus. The specific virus of different venereal diseases may be mixed in a single lesion, and thus disguise the peculiarities distinguishing one from the other of the forms of initial lesion. Great caution and repeated observations are necessary to avoid an error in diagnosis. There is no connection between gonorrhea, and chancroid, or chancre; only that there is a similarity in the mode of conveyance from one person to another. Authors speak of chancroid and chancre being conveyed from one to another, without the person conveying having any evidence of disease.

Dr. JOHNSON said that Dr. Miner's case was interesting because unusual. He saw, three weeks ago, a case where there existed a triple lesion, viz: gonorrhœa, chancroid, and chancre. The patient had also a bubo. Secondary symptoms may supervene, but not as the result of this infection, as the patient had, two years before, contracted true syphilis. There is a good deal of difference between chancre and chancroid. He had never known secondary symptoms to follow a chancroid.

Dr. GOULD. It would be safer to treat the case as syphilis.

Dr. JOHNSON. Yes, in this case, but not where there exists simply a chancroid. There is generally too much treatment for chancre—too much use of caustic. He used chemically pure nitric acid, but had not much belief in local treatment at all, unless resorted to very early.

Dr. BARNES said that all had noticed the great difference which exists between cases of gonorrhœa; often between successive attacks of gonorrhœa occurring in the same person. This difference was in the intensity of the inflammation, in the character of the discharge, being thick and purulent or thin and watery, and in the obstinacy with which cases resist treatment. It was not always that cases attended with the most acute symptoms, at first, were the most difficult to cure. These facts have given rise to the question whether gonorrhœa does not arise from many sources. While some deny, in all cases the existence of a specific poison, Hammond, in his little work on venereal diseases, does not consider as gonorrhœal, those discharges arising from causes non-specific in character, as from a leucorrhœa, excesses, etc., but asserts and claims to have proved that all cases of true gonorrhœa are caused by contact of an unbroken mucous surface, either with the virus of a soft chancre, or with that of a hard chancre, or with a discharge which though having been repeatedly transmitted, yet had originated from one of these two sources. He states that a gonorrhœa caused by contact with an indurated chancre may be followed by symptoms of secondary disease; that it has a period of incubation; yet that it is a less severe local affection, less apt to be complicated by extension of the inflammation, and less apt to pass into gleet, than that caused by a soft chancre. The latter has not a well defined period of incubation, the discharge appearing by the second or third day. Simple urethretis he considers of little moment. Dr. B. would like to hear opinions on this view of the origin of gonorrhœa.

Dr. MINER said that he had seen some suggestions of that sort. Possibly gonorrhœa is sometimes associated with a specific sore within the urethra. Gonorrhœa varies from non-specific irritation to the most acute urethretis, extending to the testicles and base of the bladder. That specific syphilitic virus ever originates from gonorrhœa he did not believe. If the patient had gonorrhœa, he was also exposed to the syphilitic lesion, and was poisoned without his knowledge.

Returning to the subject of syphilis Dr. M. said the distinguishing marks of the initial lesion were sometimes obscure. Chancroid has an incubation of from forty-eight hours to six days. Chancre, of say twenty-four days. One attack of true syphilis protects from a second as well as small pox protects from its own recurrence. Objection has been made to the early administration of mercury.

He had seen it produce a mercuro-syphilitic eruption difficult to cure, and thought it might be an open question. If there is any doubt as to the nature of the case, wait. The treatment of syphilis has been reduced to a system in works on that subject, and mercury is regarded as almost a specific in secondary. If we give up faith in mercury we must renounce faith in all medication. Nothing is more certain than its beneficial effect in the secondary stage; nothing is more marked than the benign influence of iodide of potassium after the expiration of one year. The latter remedy is useless in the primary and secondary stages, unless when the secondary is merging into the tertiary stage.

Dr. BOARDMAN would ask as to the best form for the administration of mercury. Agnew prefers inunction with mercurial ointment, applying it once or twice a day to the inner portion of the arm or of the thigh.

Dr. MINER replied that in all cases where the stomach is irritated by the internal administration of mercury, inunction is useful; so also are mercurial baths. But in his experience not much is gained by their use, except in cases of intractable skin diseases. Mercury in every form almost, is a specific. He knew of no preparation better than blue mass, none more safe, efficient and comfortable, or more apt to agree with the stomach. In the later stages, after one year, if you want to give a little mercury, use the protoiodide, or iodide of potassium with mercury. Iodine may counteract, to some extent, the effect of mercury, but if it does it is not incompatable with good treatment to give them together. Tonics are often useful.

Dr. WYCKOFF. Mercurial inunction is used on account of its mild action, and because it is less liable to produce ptyalism. It is especially useful in the later stages. He had also derived advantage from the use of mercurial baths.

It is true that cases of secondary syphilis occur without the knowledge by the patient of a primary lesion. The worst case he had ever had was an instance of this.

Dr. BOARDMAN had lately met two or three cases which had been under the treatment of Agnew and others, and in which inunction had been used. He inferred that it was a favorite form for use after the primary and early secondary stages were passed. One person who had no reason for concealment, did not know of ever having had the primary lesion.

Dr. BARNES would like to ask if it would be proper to use mercury in a person having a strumous diathesis.

Dr. MINER. Yes, patients will grow fleshy under its proper use although strumous. Even where there is tuberculosis with syphilis the best tonic is mercury. He had seen patients suffering from this combination, rapidly improve under its use.

Dr. BARNES. Is it advisable to intermit its use at intervals?

Dr. MINER. The best authorities use it a year, but not to salivation. It is often best to intermit if the gums become affected, or there is other evidence of excessive influence on the general system.

Dr. BOARDMAN. Is it known when the disease is cured; or is there any constant sign of the existence of the disease, after its apparent disappearance? It was thought that the post cervical glands would be found enlarged, but often they are not. In his examinations for the army, there were many applicants having a syphilitic taint. Some he could recognize by the smell. Others presented no symptoms by the skin, throat, or otherwise, except the condition of the inguinal glands. These were not enlarged, perhaps contracted, but were small and hard like shot, and from a line to a line and a half in diameter. He regarded this as a certain sign of a syphilitic taint in the system.

Dr. MINER. We can not always tell when a patient is cured. If a patient describes truly what symptoms he has had, we can generally tell if he has had the pox. His simple statement that he has had syphilis is not proof. We can not always ascertain the existence of a syphilitic taint. Dr. M. cited the case of a highly respectable gentleman, in whom there was not the slightest suspicion of such taint. He was sickly, pale, anæmic, etc. Dr. White gave him iron, wine, eggs and milk. It occurred to Dr. M. that he might be suffering from tertiary syphilis. He gave iodide of potassium and cured the patient. Did iodide of potassium relieve him of anæmia, or cure tertiary syphilis?

Dr. WYCKOFF. Are patients ever perfectly cured?

Dr. BOARDMAN had held that belief, and Dr. Frank Hamilton also. But now he had a case where there had been a sore twenty-two years ago, and no trouble until a year before consulting Dr. B., for a sore on the face which had been treated for cancer. After examining the groin Dr. B. accused him of having syphilis, and he admitted having had a sore at the time mentioned. Acton mentions the case of an officer who developed syphilis thirty years after the existence of the primary sore.

Dr. WYCKOFF often thought patients cured, but symptoms would afterwards recur. He doubted whether we may ever consider a patient cured.

Under prevailing diseases, Dr. BARTLETT reported scarlet fever and typhoid fever. The last was becoming more serious. It had before been mild.

Dr. BOARDMAN reported no scarlet fever, no whooping cough, one case of pure typhoid fever; also several cases taking a typhoid cast, which, in two or three weeks, would assume a distinctly malarial character, with chills, fever and sweating.

On motion Dr. Brush was appointed to read a paper on syphilis at the next meeting.

On motion the Society adjourned.

